# Effects of strength training in fibromyalgia on balance, neuromuscular performance, and symptomatic analysis: a 12-week study protocol

**DOI:** 10.3389/fneur.2023.1149268

**Published:** 2023-04-28

**Authors:** Maria Luiza L. Albuquerque, Diogo Monteiro, Marcos C. Alvarez, Guilherme Torres Vilarino, Alexandro Andrade, Henrique P. Neiva

**Affiliations:** ^1^Department of Sport Sciences, University of Beira Interior, Covilhã, Portugal; ^2^Research Center in Sports Sciences, Health Sciences and Human Development, CIDESD, Covilhã, Portugal; ^3^ESECS - Polytechnique of Leiria, Leiria, Portugal; ^4^Laboratory of Sport and Exercise Psychology, Human Movement Sciences Graduate Program, College of Health and Sport Science, Santa Catarina State University (UDESC), Florianópolis, Brazil

**Keywords:** exercise training, rheumatic diseases, pain, fatigue, anxiety

## Abstract

Fibromyalgia affects skeletal muscles presenting with muscle stiffness, pain, and fatigue. The exercise practice is wellstabilized and suggested for the reduction of symptoms. However, there are some gaps in the literature related to balance and neuromuscular performance in a strength training protocol. The aim of this study is to structure a protocol to verify the effects of strength training applied over a short period, on balance, neuromuscular performance, and fibromyalgia symptoms. Additionally, we intend to analyze the effects of a short period of cessation of training. Participants will be recruited through flyers, internet advertisements, clinical referrals, health professionals, and email outreach. Volunteers will be randomly assigned to the control group or experimental group. Before the training period, symptoms (Fibromyalgia Impact Questionnaire, Visual Analog Scale), balance (force plate), and neuromuscular performance (medicine ball throw and vertical jump) will be assessed. Participants in the experimental group will perform strength training twice a week (50 min each), on alternate days, for 8 weeks (16 sessions). Then, 4 weeks of detraining will be completed. This training program will be carried out online, using real-time videos, and the participants will be divided into two groups with different schedules. The Borg scale will be used to monitor perceived effort in each session. There is a gap in the literature about exercise prescriptions for fibromyalgia. The supervised online intervention enables a wide range of participation. The strength exercises used (without the use of external materials and/or machines) and load (few repetitions per set) represent a novelty to training programming. Moreover, this training program respects the limitations and individuality of the volunteers, providing exercise adaptations. With positive results, the present protocol could be an easy-to-apply guideline with clear instructions on the details related to exercise prescription. Also, ensure the effectiveness of a low-cost and feasible treatment, especially for fibromyalgia patients.

**Clinical trial registration:**

https://clinicaltrials.gov/, identifier: NCT05646641.

## 1. Introduction

Described in the mid-twentieth century, fibromyalgia is considered a chronic rheumatological disease (1–3) and is included in the International Classification of Diseases (ICD) since 1994. The symptoms usually associated with fibromyalgia are generalized muscle pain, fatigue, sleeping problems, anxiety, depression, headaches, balance impairments, and memory and concentration changes (4, 5). Nevertheless, the diagnosis of fibromyalgia is based on the American College of Rheumatology and criteria are primarily composed of the widespread pain index and the symptom severity scale (6). It is known that the prevalence of fibromyalgia affects around 7% of the world population (7), and it seems to be age and sex-related and varied among countries (8, 9). Indeed, fibromyalgia tended to affect mainly women in any age group, but with more emphasis when above 50 years old (7, 9). This way, it is largely seen as an actual health issue that should be further understood regarding the diagnosis and the efficacy of different strategies that can be used to improve the health and quality of life of people affected.

Fibromyalgia has been associated with alterations in the central and peripheral nervous system that can cause hypersensitivity and allodynia (10, 11). The autonomic nervous system is influenced, modifying the rates of inflammatory mediators (12). As fibromyalgia especially affects somatic tissues, the musculoskeletal system may be the most affected, presenting muscle stiffness, pain, and fatigue (6, 13). Considering these effects, the interest in physical exercise as a co-adjuvant in the treatment and improvement of the quality of life of people with fibromyalgia has grown (14). Considering the benefits of exercise in increasing muscle strength (15), improving balance (16), reducing pain (17), quality of life (18), inflammatory markers (19, 20), among other symptoms and (21), this practice has become well established in the literature. Many studies have shown positive effects of aerobic, strength, resistance and mixed exercises on physical function, fatigue, pain, and muscle strength (4, 14, 22, 23). The effects of isolated practices and specific modalities such as Pilates, Yoga, among others, are currently being sought in the literature (14, 24–27).

It is known that neuromuscular alterations caused by fibromyalgia can cause a risk of disability, as well as an increased risk of falls and injuries (28–30). For this reason, among the different training programs that were assessed, muscle strength training may be essential in combating or even preventing the onset of such issues. However, there is still weak evidence and some studies have focused on the effects of strength training on this population. Indeed, there are still some gaps in the literature regarding the training type and other variables, such as intensity and duration of practice (14, 31–33). Furthermore, the pandemic period in recent years increased the moments of social isolation and mobility restriction, perhaps contributing to accentuating the few exercise habits in this population. At the same time, this created an opportunity to develop and investigate new interventions that are suitable for this new scenario, allowing a safe and viable treatment alternative for this population (e.g., online practice). Most studies with exercise in patients with fibromyalgia are carried out face-to-face. Therefore, this randomized controlled trial protocol aims to structure a study protocol to verify the effects of strength training without external material that will be applied online over a short period (8 weeks), on balance, neuromuscular performance, and symptomatology of fibromyalgia. It is expected that the protocol can be used as a basis and/or an initial protocol for future interventions to clarify and assist the effects of fibromyalgia.

## 2. Materials and methods

### 2.1. Study design

**Figure 1:**
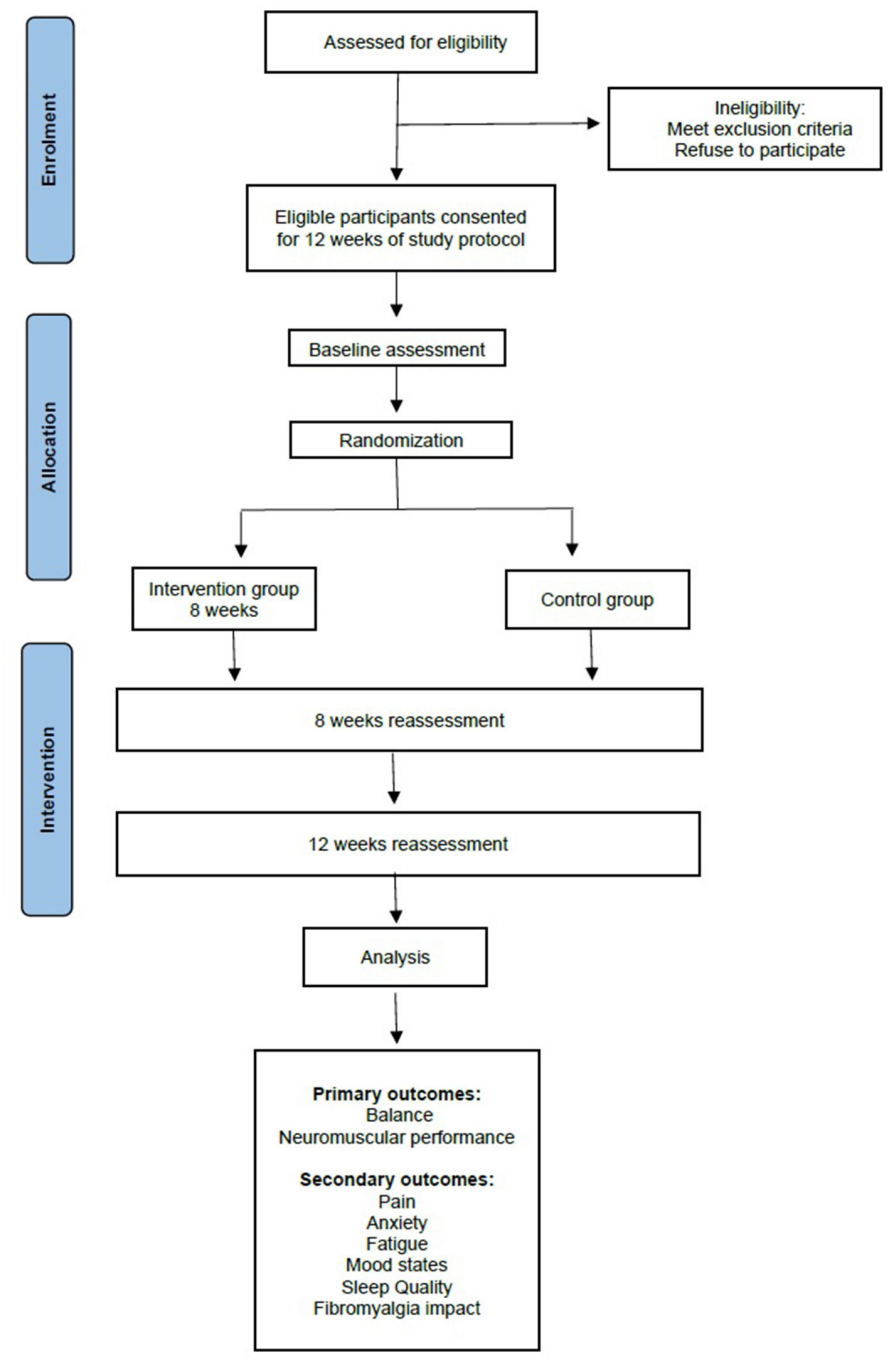
Flow diagram of the study.

The present study protocol refers to a single-blind, randomized controlled trial, developed according to Standard Protocol Items: Recommendations for Interventional Trials (SPIRIT) (34). The volunteers will be allocated randomly to the intervention group that will receive the exercise program or the control group. A staff member not involved in the study will be responsible for the randomization based on a computer-generated random number. The intervention program will last 12 weeks, consisting of 8 weeks of strength training plus 4 weeks of no training for the intervention group. The 1-month period will serve to evaluate how long the effects of the training will still be present after the training has ceased. The flow chart of the study design is represented in Figure 1.

### 2.2. Participants

The project will be publicized to find volunteers for the research. For this action, strategies such as flyers, web advertisements, clinical and health professional referrals, email disclosures, and contact from the fibromyalgia associations database will be used for participant recruitment.

After this initial recruitment, participants of both genders with a medical diagnosis of fibromyalgia according to the American College of Rheumatology criteria aged ≥18 years will be eligible to be included in the study. As exclusion criteria, it would be defined as severe comorbidity or any other type of condition that negatively influences participation in the training program; cognitive disorders; heart problems; surgeries or fractures in the last 6 months; regular practice of physical exercises in the least 3 months before the program. Will be discontinued from the study participants that: 1. change medication or dosage during the program; 2. start a regular practice of physical exercises in parallel to the training program; 3. If the participant request. Before the beginning of the program, the participants will be carefully informed about the research, and informed consent must be given before the beginning of the training program. The present research project was approved by the Ethics Committee of the University of Beira Interior, Portugal (CE-UBI-Pj-2021-017).

### 2.3. Research team

The research team will include 4 participants. Two researchers will be responsible for the evaluation of the groups—the control group and experimental group; one researcher responsible for the experimental group instruction; one staff member that is not related to the study to perform the randomization; one researcher will perform the data analysis.

### 2.4. Randomization

After evaluation of the inclusion and exclusion criteria, the participants eligible to participate in the study will be randomly separated into two groups: the control group (CG): which will have no activities performed; the experimental group (EG): which will follow the strength exercises protocol.

The randomization of the participants will be performed by an individual not directly related to the study. To avoid selection bias, random numbers will be generated by computer; the distribution will be made from a website: www.randomization.com. For the statistical analysis, the researcher in charge will receive the data without identifying the participants to obtain the results in a blinded manner. A randomization with a 1:1 computer-generated will be made for the group allocation.

### 2.5. Data collection and procedures

The collection will take place in the facilities of the institutions related to fibromyalgia closest to the participants. If the volunteer cannot attend, the evaluation will be conducted in a public and neutral environment near the region where the research participant lives.

**Figure 2:**
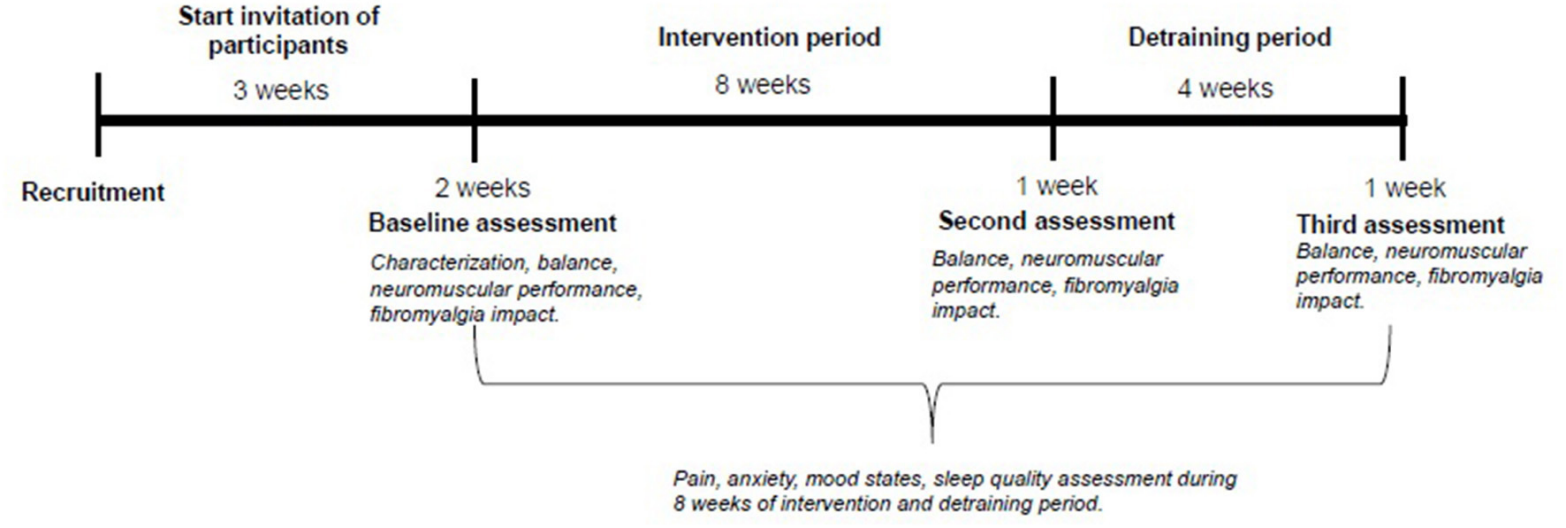
Schematic diagram of steps and procedures.

After the training period (8 weeks), the following week the participants of both EG and CG groups will be re-evaluated. From the 9th week on, the beginning of the off-training period will be accounted for, which will last 4 weeks. After this time, there will be a new reevaluation of both groups for future comparison. A schematic diagram of the schedule and steps is shown in Figure 2.

### 2.6. Outcome measures

The tests regarding the evaluation will be performed in one moment. First, demographic, and anthropometric data will be collected, followed by the Fibromyalgia impact questionnaire (FIQ-P), symptoms and balance evaluation. Neuromuscular performance data will be collected at the end so as not to influence the symptoms.

For descriptive data, the following information will be collected: sociodemographic data—age, gender, occupation, region address, marital status, history of falls (35), and due to the actual scenario of COVID-19, if there was contagion and the date of contagion will also be collected. Anthropometric measures such as weight (kg) and height (cm) will be measured using a bioimpedance scale and a stadiometer, respectively. Body mass index (BMI) will be calculated as weight (kg) divided by height (m) squared. All variables and information about the instruments are summarized in Table 1.

**Table 1:** Variables, instruments, interpretation, and reference for assessment.

**Variables**	**Instruments**	**Interpretation of instruments**	**Author and Year**
Functional capacity	FIQ-P	Scores vary from 0 to 100. Higher the value, the greater the impact of fibromyalgia.	Rosado et al. (36)
Pain intensity	VAS	Scores from 0 to 10. Higher the number, the higher intensity.	McCormack et al. (37)
Anxiety level	VAS	Scores from 0 to 10. Higher the number, the higher intensity.	McCormack et al. (37)
Fatigue level	VAS	Scores from 0 to 10. Higher the number, the higher intensity.	McCormack et al. (37)
Mood	VAS	Scores from 0 to 10. Higher the number, better state of mood.	McCormack et al. (37)
Quality of sleep	VAS	Scores from 0 to 10. Higher the number, the worse quality of sleep.	McCormack et al. (37)
Neuromuscular performance	Vertical Jump	Optical measurement system consisting of two transmitting and receiving cells. Higher jump, better neuromuscular performance.	Gonçalves et al. (41)
	Medicine Ball Throw	2kg medicinal ball throw. Higher distance, better neuromuscular performance.	Gonçalves et al. (41)
Balance	Force plate	Lower variation of the pressure centers, better balance.	Sempere-Rubio et al. (42)

FIQ-P, Fibromyalgia Impact Questionnaire Portuguese version; VAS, Visual Analog Scale.

#### 2.6.1. Fibromyalgia impact questionnaire

The translated and validated Portuguese version (FIQ-P) of the Fibromyalgia Impact Questionnaire (36) will be used to access information on the functional capacity and health status of individuals with fibromyalgia. This questionnaire is composed of 20 questions, divided into 10 items that address physical functioning, work status, depression, anxiety, sleep, pain, stiffness, fatigue, and wellbeing.

The item referring to functional ability is the most extensive, which is divided into 11 sub-items, referring to the activities of daily living. The answers are answered on a Likert-type scale from 0 (always able to perform) to 3 (unable to perform). To obtain the results of this item, it is necessary to sum the answers and divide them by the number of valid questions. The next two items refer to the days of the week, being chosen from a number from 1 to 7-questions 12 and 13. For the remaining items 14 to 20, the visual analog scale (VAS)-0 to 10 (maximum value) is used. After obtaining the results, the values vary from 0 to 100, in which the higher the value, the greater the impact of fibromyalgia.

#### 2.6.2. Visual analog scale

For the evaluation of the other symptoms related to fibromyalgia, for the present study, the Visual Analog Scale (VAS) (37) was chosen for the analysis of pain intensity, anxiety level, fatigue level, mood, and quality of sleep. This scale consists of a 100-millimeter horizontal line with boundaries at each end, in which the final rating is measured from the distance from the initial end to the mark by the participant. In this way, each millimeter will be equivalent to one unit. The scoring range will be from 0 to 100, in which the higher the value, the greater the intensity. Scores between 0 and 30 mm will be classified as “mild pain”; between 40 and 70 mm as “moderate pain” and between 80 and 100 mm as “severe pain.”

#### 2.6.3. Fibrodaily

Data regarding pain intensity, anxiety level, fatigue level, mood, and quality of sleep will be continuously collected. A cell phone application will be developed, in which the participants will answer daily the parameters mentioned above. All the outcomes will be defined from VAS with scores from 0 to 10. Zero will constitute the inexistence of pain, anxiety, or fatigue, while intensity will be graded from 1 (mild) to 10 (extremely intense). The outcomes of sleep quality and mood, the scale will be from 0 (bad/not cheerful) to 10 (excellent/extremely excited). To minimize the limitation of poor technology skills, support will be offered to participants. The volunteer will receive a paper spreadsheet to register daily symptoms in case of incompatibility or error during the application's use.

#### 2.6.4. Neuromuscular performance

To assess neuromuscular performance two different tests will be performed. The vertical jump protocol evaluates the neuromuscular performance of the lower limbs, while for the upper limbs, it will be used the Medicine Ball Throw test (38, 39). Before the assessments, a general and specific warm-up of 10 min will be performed (40).

##### 2.6.4.1. Vertical jump

The participants will initially perform vertical jumps, and the heights will be obtained in two different moments: the countermovement jump (CMJ) and the countermovement jump with free arms (CMJFA). Each test will consist of 3 jumps with 2 mins rest between each attempt. The starting position for the CMJ test will be with the hands on the hips and knees fully extended. For the MCJF test, the knees should be extended, but flexion and extension of the upper limbs during the jump will be allowed. During both jumps, volunteers will be instructed to descend to personal counter-movement depth and jump as high and fast as possible. To obtain these data, an optical measurement system consisting of two transmitting and receiving cells (Optojump Next; microgate, Bolzano, Italy) will be used. For performance evaluation and calculation of results, a protocol developed and previously executed will be followed (41).

##### 2.6.4.2. Medicine ball throw

This test aims to evaluate the strength of the upper limbs, more specifically in this case, horizontally. The evaluation will be performed individually with three attempts to throw a 2 kg medicinal ball with 1-min rest between each throw. The other performance criteria will follow a previously published protocol (41). The objective of the test is to quantify the distances obtained from the throw performed by the individual. Measures of reliability (Intraclass Correlation Coefficient and coefficient of variation) will be calculated, in addition to using the mean and maximum value obtained in the trials.

#### 2.6.5. Balance

To assess the balance, a force plate (MuscleLab, Ergotest Innovation, Porsgrunn, Norway) will be used. It will be possible to identify the variation of the pressure centers in the different stimuli or sensorial alterations. A previously used protocol for this evaluation will be used as the basis for the present study (42). The tests will be evaluated following the previously cited protocol: 1. standing position with eyes open (EO); 2. standing position while recalling a stressful day (Dual task [DT]) and standing position with eyes closed (EC).

### 2.7. Intervention

#### 2.7.1. Experimental group

To include participants from all over the country, the training program will be carried out through real-time videos. The protocol consists of 8 weeks of training, consisting of 16 sessions of 50 mins, twice a week on alternate days. To facilitate follow-up during practice and reduce the risk of dropout, the volunteers will be divided into two groups with different times for each intervention. All volunteers will perform the exercise protocol at the meeting via an online (Google Meet platform) and both interventions will be performed during the same shift of the day. Each group will consist of 6 to 8 online participants. The sessions will be conducted and supervised by a physical therapist. Volunteers will be instructed about the exercises, protection and how to use the Borg scale at beginning of the meeting. During the meeting, before each exercise, the movement will first be demonstrated by the physical therapist, and then, sets and repetitions will be informed. To ensure that each participant will do the same number of sets and repetitions, all participants will perform the movement during the professional's count. This format will make it easier to count the rest time.

The program will consist of 10 mins of warm-up, 35 mins of strength exercises, and 5 mins of cool-down. The Borg scale will be used to monitor fatigue and perceived effort during practice (43). The protocol of the exercises to be performed during the training program, as well as their progression, are described in Tables 2, 3, respectively.

**Table 2:** Description of strength training.

**Exercise**	**Name**	**Description**	**Position**	**Muscles**
Exercise 1	Sumô Box Squat	Stand near the chair with your feet wider than shoulder width. Start the movement standing with your hips and sit back. Be careful with the chest, knees, and shins.	Stand	**Primary:** Quadriceps. **Secondary:** Gluteus maximus, medius, and minimus; hamstrings, adductor magnus, longus, and brevis; erector spinae.
Exercise 2	T-exercise	From a standing position, bend at the hips and maintain a neutral spine. Perform dynamic motions by forming a T with the arms and return to starting position.	Stand	**Primary:** Lower and middle trapezius; rotator cuff musculature; posterior deltoid. **Secondary:** Hamstrings; gluteus maximus.
Exercise 3	Torso-elevated push-up	Place your hands on the wall wider than shoulder width with the feet closer together on the ground. Keep the body in a straight line and lower the body until the chest touches the wall. Reverse the movement and raise the body. Be careful with the lumbar spine and elbows.	Stand	**Primary:** Pectoralis major; triceps brachii; anterior deltoid. **Secondary:** Serratus anterior; trapezius; rectus abdominis.
Exercise 4	Bird dog	On quadruped position with the spine in a neutral position, the hands under the shoulders and the knees under the hips. Kick one leg back until the full extension is reached adding an opposite diagonal upper-body movement. Return to starting position.	Quadruped	**Primary:** Gluteus maximus; pectoralis major and anterior deltoid. **Secondary:** Hamstrings; erector spinae; adductor magnus, longus and brevis; gluteus medius, and minimus; multifidus.
Exercise 5	Bridge	In dorsal decubitus keep the knees bent at 90 degrees. Push through the heels and raise the hip. Hold the position in the top for a few seconds and lower the hip to starting position.	*Dorsal Decubitus*.	**Primary:** Gluteus maximus. **Secondary:** Hamstrings; erector spinae; adductor magnus, longus and brevis; gluteus medius and minimus.
Exercise 6	Floor fly	In dorsal decubitus keep the knees bent at 90 degrees. With palms facing, lower the arms in an arching motion. When your elbows hit the floor, back together in an arching motion.	*Dorsal Decubitus*.	**Primary:** Pectoralis major, anterior deltoid. **Secondary:** biceps brachii.
Exercise 7	Single-leg up and down	In dorsal decubitus keep one knee bent with one foot planted on the ground. Keep the opposite hip and knee extended. Up the straight leg until align with the opposite knee. After the movement, return to starting position. Be careful with the lumbar spine.	*Dorsal Decubitus*.	**Primary**: Lower rectus abdominis; psoas major; rectus femoris. **Secondary:** Upper rectus abdominis; internal and oblique.

**Table 3:** Training progression during the program.

**Weeks**	**Sets**	**Repetitions**	**Intensity**	**Rest**	**Perceived effort**
1–2	3	8	Maximal intended velocity^*^	1 min and 30 secs	12–13 RPE
3–4	3	8		1 min and 30 secs	12–13 RPE
5–6	5	6		1 min and 30 secs	14–16 RPE
7–8	5	6		1 min and 30 secs	14–16 RPE

RPE, rate of perceived exertion.

^*^The concentric phase of the movement will be as fast as possible.

The training will include 3–5 sets of 6–8 repetitions depending on the week, as listed in Table 3. The evolution of the training program will be done according to the individuality of each participant. During the protocol, in case of signs of extreme fatigue, reduced range of motion, or inability to perform the movement, an adaptation of the exercise will be made. Table 4 shows the adaptation for each previously defined exercise. Any adjustment will be performed by changing the exercise and/or rest interval. All exercises must be performed with the highest quality, which means technically correct performance and maximum range of motion. Furthermore, the concentric phase should be performed as fast as possible, at the maximal intended velocity.

**Table 4:** Exercise adaptations.

**Exercise**	**Easier adaptation**
Sumô Box Squat	Box squat
T-exercise	Perform extension of the upper limbs with flexion of the elbows
Torso-elevated push-up	Decrease body inclination
Bird dog	Keep the hands on the floor and keep the movement with the legs
Bridge	Decrease the height of the hip lift
Floor fly	Increase elbow flexion during the movement
Single-leg up and down	Decrease the height of the leg lift

#### 2.7.2. Control group

Participants included in the control group in a randomized manner will be instructed to maintain their usual medical treatment and daily routines during the experimental intervention period, which did not include structured exercise. After the end of the protocol, the patients were re-evaluated and invited to receive the training program. After the end of the protocol and the re-evaluation, control group participants will be given the opportunity to perform the training program.

### 2.8. Retention and adherence

To maintain adherence, individualized attention will be prioritized during the session and especially the care regarding pain control of everyone at the beginning, during and at the end of each session. In case of absence, the participant will be contacted for information regarding his/her health status or other reasons. In case of withdrawal from the program, the reason will be investigated to be identified at the end of the study.

### 2.9. Sample size calculation

The G-power software was used to detect a moderate effect size (partial eta squared of 0.09) in the intra/inter-group comparison. Considering an alpha value of 5%, the total number of participants should be 24 so that the power of the results is 90%. Considering a 20% of dropout risk, a minimum of 30 participants will be recruited (15 for each group).

### 2.10. Statistical analysis

SPSS software will be used. To verify normality either the Shapiro-Wilk test (n < 30) will be used, and the equality of variances will be assessed by the Levene test. If the data, follow a normal distribution inter-group and intra-group differences will be calculated by repeated measures analysis of variance (ANOVA). If the data do not follow a normal distribution, the Kruskal-Wallis test will be calculated for between-group differences and the Friedman test for intra-group differences. To calculate the effect size, Cohen's test (intra-group comparison) will be used, and the between-group variance (partial eta squared) will be estimated. To verify the reliability of the tests and their measurements, the coefficient of variation and the intraclass correlation coefficient will be calculated. Finally, to evaluate the relationships between variables, Pearson's correlation coefficient will be calculated. The significance level will be set at 0.05.

## 3. Discussion

Since the last decade, has been a growing number of investigations related to fibromyalgia and specifically the relation of the syndrome with physical exercise (23, 32, 44, 45). Previous studies have already proven the effectiveness of different types of exercise on symptoms such as quality of life, anxiety, and balance, among other symptoms (21, 46). Despite such findings, most studies were conducted before the Covid-19 pandemic. The compliance rate for exercise by fibromyalgia patients before the pandemic was only 57.7% (47). During the social isolation caused by Covid-19, only 28.1% remained active during quarantine and the dropout rate of exercise programs was 46.7% (47). The lockdown had a negative impact on mental health and pain (48). The lack of motivation or worsening symptoms were already considered barriers to exercise before the pandemic (49). These factors were amplified during this time and could be related to the dropout or reduction of physical activity during this period. Furthermore, the impossibility of access to a specific place to perform physical activities during this time might have been another important contribution to dropout (47).

Given the aforementioned contextual changes, there is an urgent need for the encouragement of physical exercise, perhaps studying different forms of conducting and following physical exercise programs. Supervised online practice can be an interesting alternative as a form of support for these individuals. In addition, strength training ends up being less studied when compared to aerobic-type modalities, and more studies are needed in the area to obtain greater relevance as to its effect (14, 32). However, due to the pandemic, adaptations to intervention studies were necessary (50). Studies adapted virtual format have achieved positive results in disease impact with symptom reduction, proving to be effective and accessible (51, 52). Performing the interventions in real-time rather than pre-recorded videos may be a helping factor in avoiding the loss of research participants.

Another novelty of this protocol is the performance of strength exercises without the use of external materials and/or machines, using fewer repetitions per set than usual. This would probably allow the participant to perform each exercise without an excessive velocity loss in each set, enabling higher velocity stimulation and less fatigue per set (53). The high movement velocity and the low-velocity loss (few repetitions per set) are believed to stimulate the amount of force produced in little time, improving the neuromuscular response of the participants, and thus helping their functional capacity (54, 55). This decision goes differently from most of the exercise protocols found in the literature (14, 33).

A possible limitation of the study will be the participants' adherence due to the online intervention. Another limitation is the impossibility of blinding the instructor since it will be necessary to perform some evaluations. The results analysis will be done by another researcher, as informed in the methods to reduce the risk of bias.

Our protocol will provide an excellent strategy for application in new public health policies. Especially for communities and countries that seek to expand the reach of physical exercises as part of the treatment actions, focused on rheumatic diseases and specifically on FM. Physical Exercise is increasingly seen as a protective factor for health. This protocol increases the chances for this health service, with recognized benefits, reaching more people and groups with specific treatment needs and increasing accessibility, as in the case of our study. It will contribute to improving the cost-benefit ratio in the implementation of treatment. Furthermore, the knowledge obtained from the implementation of this protocol would allow a step forward on the possibility of designing a multicomponent training program including other types of training (e.g., aerobic and stretching) for this population.

It is important to highlight that the hybrid approach of this protocol constitutes the innovation of the investigation. The on-site evaluations will help complement the data for further support of the existing gap in the literature regarding balance and neuromuscular performance through a strength protocol. The online intervention will allow us to evaluate the participants' adherence and evolution regularly. It is expected to achieve positive results through a new approach to the strength training protocol that respects the limitations and individuality of the volunteers with adaptations of the exercises. Based on such results, the program will aim to increase independence by performing the exercises at home and perhaps contribute to sedentarism reduction. An exercise protocol that encourages the initiation of physical exercise is of utmost importance to assist in the management of fibromyalgia. Furthermore, the present protocol is easy to apply and has clear instructions and details related to exercise prescription.

## Ethics statement

The studies involving human participants were reviewed and approved by Ethics Committee of the University of Beira Interior, Portugal (CE-UBI-Pj-2021-017). The patients/participants provided their written informed consent to participate in this study.

## Author contributions

MLLA and HN: conceptualization and writing. DM and MA: review. AA and GV: writing—review. HN: review and supervision. All authors have contributed significantly to this manuscript and agreed with its content and take full responsibility for the integrity of the study and the final manuscript.
